# Tracking data from nine free-roaming Cheetahs (*Acinonyx jubatus*) collared in the Thabazimbi area, Limpopo Province, South Africa

**DOI:** 10.3897/BDJ.5.e11323

**Published:** 2017-02-23

**Authors:** Kelly Marnewick, Samantha Page-Nicholson, Lizanne Roxburgh, Michael J. Somers

**Affiliations:** 1 Endangered Wildlife Trust, Johannesburg, South Africa; 2 Centre for Wildlife Management, University of Pretoria, Pretoria, South Africa

**Keywords:** Satellite tracking, free-roaming Cheetah, conservation, spatial ecology, outside of protected areas

## Abstract

**Background:**

In partnership with the University of Pretoria, the Endangered Wildlife Trust’s Carnivore Conservation Programme collared six male and three female free-roaming Cheetahs (*Acinonyx jubatus*) in the Thabazimbi area in Limpopo Province, South Africa. This study was undertaken to determine the spatial ecology of free-roaming Cheetahs that occur outside of formal protected areas on private ranchland, where they frequently come into conflict with, and are sometimes killed by, private landowners. The data were collected between September 2003 and November 2008, resulting in a total of 3165 location points (65 points from VHF collars and 3100 from GPS collars) for nine individual Cheetahs.

**New information:**

This dataset provides distribution information about this Vulnerable species occurring outside of protected areas within South Africa. The dataset has been published to the Global Biodiversity Information Facility (www.GBIF.org) and provides the largest dataset on Cheetahs thus far, and, although it is spatially limited to a relatively small region on the African continent, it is the first study of its kind within South Africa. Also of significance is that the fate of 6 of the 9 collared Cheetahs is known, all except one of which died of anthropogenic causes.

## Introduction

Three populations of the Vulnerable Cheetah (*Acinonyx jubatus*) exist in South Africa: 1) a population of approximately 400 individuals occurring in the Kruger National Park (Marnewick et al. 2014), 2) a metapopulation that requires intensive management, comprising of approximately 300 Cheetah which are temporarily in private or state protected areas (van der Merwe et al. 2016) and 3) a free-roaming population of unknown size occurring on private ranchland outside of protected areas (Marnewick 2015). This population is largely under threat by human-wildlife conflict which results in the loss of cheetahs, and other predators, through retaliatory killings to protect livestock or managed game species (Marnewick and Somers 2015), as well as capture for the illegal wildlife trade (Endangered Wildlife Trust, unpublished data).

This free-roaming population (which is a naturally occurring population where the movement of individuals is not restricted by fences or management) is important because, in South Africa, the protected area network alone is insufficient for conserving populations of large carnivores (Marnewick and Somers 2015). Only two protected areas in South Africa, Kruger National Park and Kgalagadi Transfrontier Park, hold substantial populations of cheetahs (Lindsey and Davies-Mostert 2009). The free-roaming population occurs along the northern borders of the country, and comprises the largest proportion of the distribution range of this species (Marnewick 2015), and thus also the largest proportion of the population, although the actual population size is unknown, but estimated with low confidence at between 400 and 800 individuals (van der Merwe et al. 2016).

Cheetahs also often transverse the porous boundaries of protected areas moving onto private lands with prey and few or no competing predators. On private land, larger carnivores such as Lion (*Panthera leo*) and Spotted Hyaenas (*Crocuta crocuta*) have been extirpated, leaving a depauperate carnivore guild dominated by Cheetahs, as well as Leopards (*Panthera pardus*) and Brown Hyaenas (*Parahyaena brunnea*). A few studies have been done to determine the movement and range use of free-roaming populations of Cheetahs (Houser et al. 2009), but this was the first study to do so in South Africa.

## General description

### Purpose

The main aim of this study (in the form of a Doctoral thesis; Marnewick 2015) was to determine, through VHF and later GPS tracking, the range use of Cheetahs outside of protected areas. This information could ultimately guide conservation action for this species, and in particular provide information that is directly relevant to landowners to use in conflict mitigation, and to address mis-perceptions about cheetah behaviour on their ranches. Data were collected between September 2003 and November 2008 after collaring of cheetahs with various tracking devices. Data are co-owned by the University of Pretoria and The Endangered Wildlife Trust. The current database is held with the EWT’s Carnivore Conservation Programme.

### Additional information

The study revealed the following key results:

- Of the 9 collared cheetahs, 4 were shot by landowners and one died in a road accident. One appeared to have died from natural causes, while the fate of the remaining 3 was unknown.
- Male Cheetah home ranges are generally larger than female ranges. Male 95UDs ranged from 121.5km^2^ to 607km^2^ while females ranged from 14.7km^2^ to 703.3km^2^ (Marnewick & Somers 2015).
- Home ranges were generally larger than the average ranch size of 18 km^2^, and cheetahs typically ranged across an area approximate in size to 18 properties (mean male muinimum convex polygon is 1597.2km^2^ while female is 698 km^2^). (Details on how home ranges were analysed and determined are available in Marnewick & Somers 2015).
- It was concluded that the large ranges of Cheetahs outside protected areas are likely driven by two key factors: 1) the search for suitable habitat in an encroached environment and 2) human avoidance (Marnewick & Somers 2015). Cheetahs in other systems (e.g. Kruger National Park) are mainly driven by alternative factors such as prey availability and competition with more dominant predators.
- Although what drives the large range use of Cheetahs in Thabazimbi could not be determined, this study provides useful information on the movement of Cheetahs and shows that generally, Cheetahs do not limit their movement to one property, thus preventing severe impacts on the prey base on individual properties Caro 1994, Marker et al. 2008).

## Project description

### Title

Tracking data from nine Cheetahs (*Acinonyx jubatus*) collared in the Thabazimbi area, Limpopo Province, South Africa.

### Personnel

Kelly Marnewick, Deon Cilliers, Luke Strugnell, Grant Beverley, Peter Caldwell

### Study area description

The Thabazimbi District in Limpopo was used as the core study area. The mean ranch size in the district is 18km^2^ (KM unpublished data) with the key land-use being wildlife ranching or a combination of wildlife and stock ranching (Wilson 2006). Properties are sporadically fenced and the fencing depends on the property land use (either standard game fencing or cattle fencing). Fences on such properties have not been found to restrict Cheetah movement. However, some properties had predator proof camps and these would restrict Cheetah movement, however was an insignificant proportion of the study area. Three main rivers flow through the area: the Marico River that forms the border with Botswana, the Matlabas River forms the approximate eastern extent of the study area, and the riparian zone of the Crocodile River is used for intensive commercial crop farming. The area is geographically uniform and topographically flat and bordered on the south and south-east by two mountain ranges, the Witfonteinrant Mountains and the Dwarsberge. The Thabazimbi District lies in the Savanna Biome of South Africa and the main vegetation type is Mixed Bushveld, and, where the soil is more clayey, Clay Thorn Bushveld (Low and Rebelo 1996). The area has been historically used for cattle ranching and the bush is encroached over a large portion of the district (KM unpublished data, pers obs.). There are some previously ploughed areas that have since been left fallow. The annual but mainly summer rainfall for the study area varies from 350 mm to 650 mm per year with temperatures ranging from -8°C to 40°C with an annual mean of 21°C (Low and Rebelo 1996). Human population density is low at 2/km^2^ (Statistics South Africa 2001). In addition to free-roaming Cheetahs there are additionally Brown Hyaenas (*Hyaena brunnea*) and Leopards (*Panthera pardus*) with occassional reports of African Wild Dog (*Lycaon pictus*). Prey in the area includes livestock and wild game (Marnewick 2015).

### Funding

Columbus Zoo, Cat Life Foundation, Duemke Family Trust, Scovill Zoo, Carston Springs Trust and the DST-NRF Centre for Excellence for Invasion Biology

## Sampling methods

### Study extent

The study covers two provinces within South Africa; the Limpopo Province and the North-West Province. Most data points (> 95%) occur in Limpopo; typically around the Thabazimbi area located in the western reaches of the province. Some of the points (< 5 %) occur in the northern areas of the North-West Province. The study comprised a component of a Doctoral thesis by Kelly Marnewick at the University of Pretoria.

### Sampling description

Cheetahs were trapped using double door traps which were placed at three various site location types: 1) using live, but protected, goats (*Capra aegagrus hircus*) as bait in areas where Cheetahs had been detected 2) on fence lines (standard game fences) frequently walked by cheetahs, and 3) at Cheetah scent marking posts (Marnewick 2015; Marnewick and Cilliers 2006). Trapped Cheetahs were immobilised by a professional wildlife veterinarian and fitted with tracking collars. In instances where coalitions were caught, only one member of the coalition or group was fitted with a tracking collar. Cheetahs were allowed to recover from immobilisation in the trap cage and were released at the site of capture. Cheetahs were monitored for the extent of their life or the life of the collar. All activities involving Cheetah handling and research were done under the guidance of the University of Pretoria Animal Use and Care committee (reference number: EC030-09) and with permits issued by Limpopo Economic Development Environment and Tourism department (the local conservation authority). Trapping success was low with approximately 278 trap days required to trap a cheetah. Cheetahs were monitored for between 28 and 2 119 days, depending on the life of the cheetah or the collar.

Initially, VHF collars (African Wildlife Tracking, Pretoria, South Africa) were fitted to two individuals. Later in the study, these were replaced by GPS/GSM collars (African Wildlife Tracking, Pretoria, South Africa & Hot Group, Pretoria, South Africa) that were utilized to obtain more robust data. The latter devices were set to take either two or four locations per day (at 12h00 and 00h00 for the collars set for two daily locations and additional times of 06h00 and 18h00 for collars with four daily locations). Two collars needed to be replaced as a result of deteriorating batteries. The two male (AM196 - GeorgeJoss) and three male (AS68 - CBU) coalitions were initially monitored using VHF collars resulting in 56 (2.8% of total) and 12 (8.6% of total) data points being obtained respectively. Locations for animals wearing VHF collars were recorded by tracking the individuals from a microlight aircraft with one pilot and one researcher on board. For GPS collars, all GPS fixes were recorded directly from the device and transmitted through the GSM cell phone network. The data were then accessed and downloaded through an online platform. See Table 1

### Quality control

The dataset has gone through a cleaning and georeference verification process to ensure GPS points and location information is accurate. Fourteen records were removed from the dataset as they were determined to be outliers due to their large distance from the study area in general, and also from other consecutive GPS locations. Terms in the dataset are in accordance with those set by the Darwin Core (DwC) Standard (Darwin Core Task Group, 2009). The dataset was published to the Global Biodiversity Information Facility (GBIF) in September 2016, and is thus freely available for download and use (see Usage Rights and Data Resources sections).

## Geographic coverage

### Description

**Figure 1.**
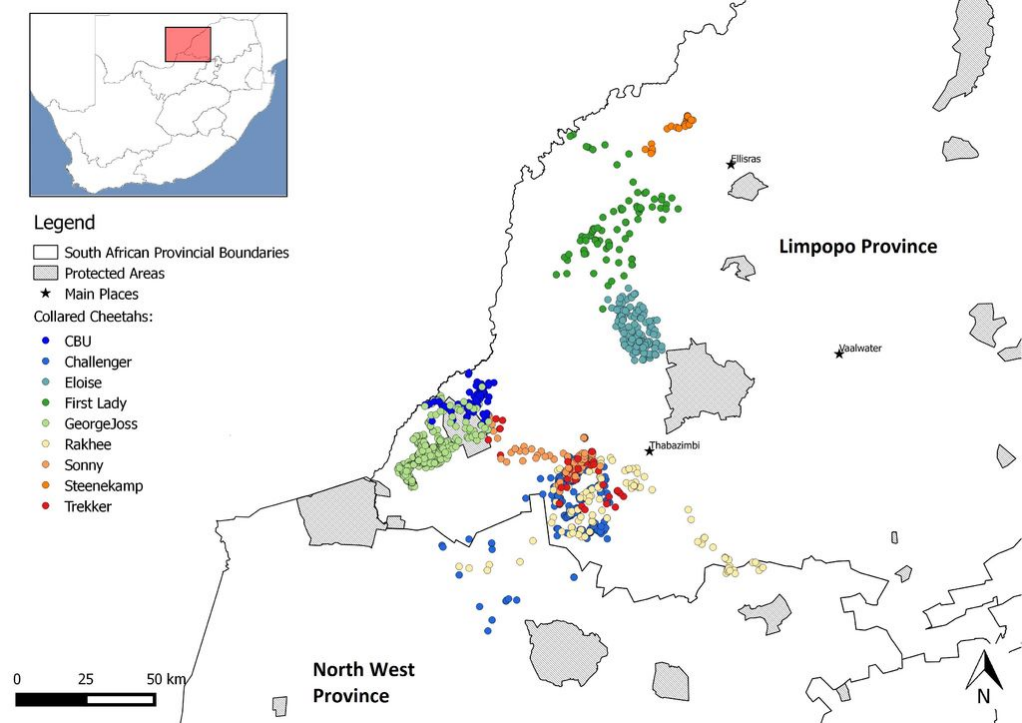
The spatial distribution of nine collared Cheetahs in South Africa between September 2003 and November 2008.

The geographic range of the bulk of the dataset covers two South African provinces: 1) the north-west reaches of Limpopo Province and 2) the northern areas of the North West Province (Fig. 1).

### Coordinates

-25.16 and -23.49 Latitude; 27.81 and 26.50 Longitude.

## Taxonomic coverage

### Description

**Kingdom**: Animalia

**Phylum**: Chordata

**Class**: Mammalia

**Order**: Carnivora

**Family**: Felidae (Cats)

**Subfamily**: Felinae

**Common Names**: Cheetah

**Genus**: *Acinonyx*

**Species**: *A. jubatus*

**General taxonomic coverage description**: This dataset focuses exclusively on Cheetah (*Acinonyx jubatus*), which is categorized as Vulnerable in the IUCN Red List (Durant et al. 2015). It belongs to the family Felidae within the order Carnivora.

### Taxa included

**Table:** 

Rank	Scientific Name	Common Name
species	*Acinonyx jubatus*	Cheetah

## Temporal coverage

**Data range:** 2003-9-18 – 2008-11-21.

### Notes

Cheetahs were trapped between September 2003 and November 2008; giving approximately five years of data and 3165 data points.

## Usage rights

### Use license

Open Data Commons Attribution License

### IP rights notes

Use of data is permitted for both profit and non-profit ventures. Data are provided to users but it may not be passed on or redistributed to third parties. The use of this data in other databases is strictly forbidden.

## Data resources

### Data package title

EWT: Carnivore Conservation Programme Cheetah Tracking Data

### Resource link

doi: 10.15468/0nsr0r

### Number of data sets

1

### Data set 1.

#### Data set name

EWT: Carnivore Conservation Programme Cheetah Tracking Data

#### Data format

DarwinCore

#### Number of columns

22

#### Download URL

http://www.gbif.org/dataset/77b04b75-97f5-4d3c-9184-8cc5c12d71ae

#### 

**Data set 1.** 

Column label	Column description
institutionCode	The name (or acronym) in use by the institution having custody of the object(s) or information referred to in the record
ownerInstitutionCode	The name (or acronym) in use by the institution having ownership of the object(s) or information referred to in the record
basisOfRecord	The specific nature of the data record. Recommended best practice is to use a controlled vocabulary such as the list of Darwin Core classes. In this case, the basis of record is either "HumanObservation", when animals wearing VHF collars were tracked and observed by human observers, or "MachineObservation", when the location of an animal wearing a GPS/ GSM collar was determined by the GPS/ GSM collar.
occurrenceID	An identifier for the Occurrence (as opposed to a particular digital record of the occurrence). Constructed from a combination of identifiers in the record that will most closely make the occurrenceID globally unique.
catalogNumber	An identifier for the record within the data set or collection
sex	The sex of the biological individual(s) represented in the Occurrence
lifeStage	The age class or life stage of the biological individual(s) at the time the Occurrence was recorded
occurrenceRemarks	Comments or notes about the Occurrence
organismID	An identifier for the Organism, which is specific to this data set only
organismName	A textual name or label assigned to an Organism instance
eventDate	The date-time or interval during which an Event occurred. For occurrences, this is the date-time when the event was recorded
eventTime	The time or interval during which an Event occurred
year	The four-digit year in which the Event occurred, according to the Common Era Calendar
month	The ordinal month in which the Event occurred
day	The integer day of the month on which the Event occurred
fieldNotes	The text of notes taken in the field about the Event. This is only relevant for those records that derive from VHF collar tracking of the individuals
decimalLatitude	The geographic latitude (in decimal degrees, using the spatial reference system given in geodeticDatum) of the geographic center of a Location. Positive values are north of the Equator, negative values are south of it. Legal values lie between -90 and 90, inclusive
decimalLongitude	The geographic longitude (in decimal degrees, using the spatial reference system given in geodeticDatum) of the geographic center of a Location. Positive values are east of the Greenwich Meridian, negative values are west of it. Legal values lie between -180 and 180, inclusive
geodeticDatum	The ellipsoid, geodetic datum, or spatial reference system (SRS) upon which the geographic coordinates given in decimalLatitude and decimalLongitude as based
scientificName	The full scientific name
family	The full scientific name of the family in which the taxon is classified
vernacularName	A common or vernacular name

## Additional information

**Papers published from the dataset**:

- Marnewick, K. & Cilliers, D. 2006. Range use of two coalitions of male cheetahs *Acinonyx jubatus* in the Thabazimbi district of the Limpopo province, South Africa. South African Journal of Wildlife Research 36 (2): 147-151.
- Marnewick, K. & Somers, M.J. 2015. Home range size of cheetahs *Acinonyx jubatus* outside protected areas in South Africa. African Journal of Wildlife Research 45 (2): 223–232.
- Marnewick, K. 2015. Conservation biology of cheetahs *Acinonyx jubatus* (Schreber, 1775) and African wild dogs Lycaon pictus (Temminck, 1820) in South Africa. Phd. University of Pretoria.

## Figures and Tables

**Table 1.** A description of the Cheetahs and collars used

**UnitID**	**individualID**	**Sex**	**DateFitted**	**LastFix**	**NumberofFixes**	**Fate**	**CollarType**
AM124	Challenger	Male	7/14/2006	3/21/2007	394	Unknown	GPS/GSM Collar
10000037	Eloise	Female	9/20/2007	4/17/2008	167	Shot	GPS/GSM Collar
AM257	First Lady	Female	5/18/2007	11/15/2007	90	Roadkill	GPS/GSM Collar
AM196	GeorgeJoss (coalition of two)	Male	9/18/2003	7/7/2009	1954	Natural Causes	VHF Collar
Rak	Rakhee	Male	8/3/2006	10/13/2006	150	Shot	GPS/GSM Collar
AM224	Trekker	Male	6/5/2007	10/16/2007	43	Unknown	GPS/GSM Collar
AS121	Sonny	Male	7/4/2008	11/21/2008	196	Unknown	GPS/GSM Collar
10000049	Steenekamp	Female	12/12/2007	1/9/2008	31	Shot	GPS/GSM Collar
AS69	CBU (coalition of three)	Male	5/3/2004	2/9/2008	140	Shot	GPS/GSM Collar & VHF Collar
